# Meckel's Diverticulum With Perforation

**DOI:** 10.7759/cureus.67026

**Published:** 2024-08-16

**Authors:** Mohamed Ahmed, Rasha Saeed, Ahmed Allawi, Jami Zajicek

**Affiliations:** 1 Surgery, University of California, Riverside, Riverside, USA; 2 Occupational Medicine/Environmental Medicine, University of California, Irvine, Irvine, USA; 3 Surgery, AdventHealth, Tampa, USA

**Keywords:** generalized abdominal pain, nausea and vomiting, ruptured appendicitis, rare cause of acute abdominal pain, perforated meckel's diverticulum

## Abstract

Meckel's diverticulum (MD) is a common congenital defect of the small intestinal tract resulting from incomplete obliteration of the vitellointestinal duct. It presents with unexplained gastrointestinal bleeding, bowel obstruction, and inflammation. In rare instances, the presentation is with perforation similar to acute appendicitis with perforation. The symptoms, clinical exam, and radiological findings of our patient, a 38-year-old male, were consistent with perforated acute appendicitis; he was found to have a perforated Meckel's diverticulum intraoperatively.

## Introduction

Meckel's diverticulum (MD) results from incomplete obliteration of the omphalomesenteric duct (Vitello intestinal duct) and is a common congenital anomaly of the small intestine [1]. During embryological development, the omphalomesenteric duct connects the yolk sac to the small intestine and provides nutrition until placenta formation accompanied by duct separation and involution around seven weeks of gestation. Partial or complete failure of obliteration can result in an omphalomesenteric cyst, fistula, or fibrous band [2]. MD was first described by Fabricus Heldanus in 1650 [3], and the embryonic origin was established by the German comparative anatomist Johann Friedrich Meckel in 1809 [4]. The rule of twos had been used to describe this anomaly, as it is twice as common in boys, two inches in length, two feet from the ileocecal valve, can contain two types of heterotropic mucosa, and has a 2% incidence rate [5]. MD is usually clinically silent and discovered during abdominal exploration and on diagnostic imaging [6]. Presentation is dependent on the underlying pathology with abdominal pain when inflamed, gastrointestinal bleeding when it harbors gastric heterotropic mucosa, or small bowel obstruction secondary to intussusception or volvulus [7]. MD perforation is rare and has an incidence of 0.5% of all presentations [8].

## Case presentation

**Figure 1:**
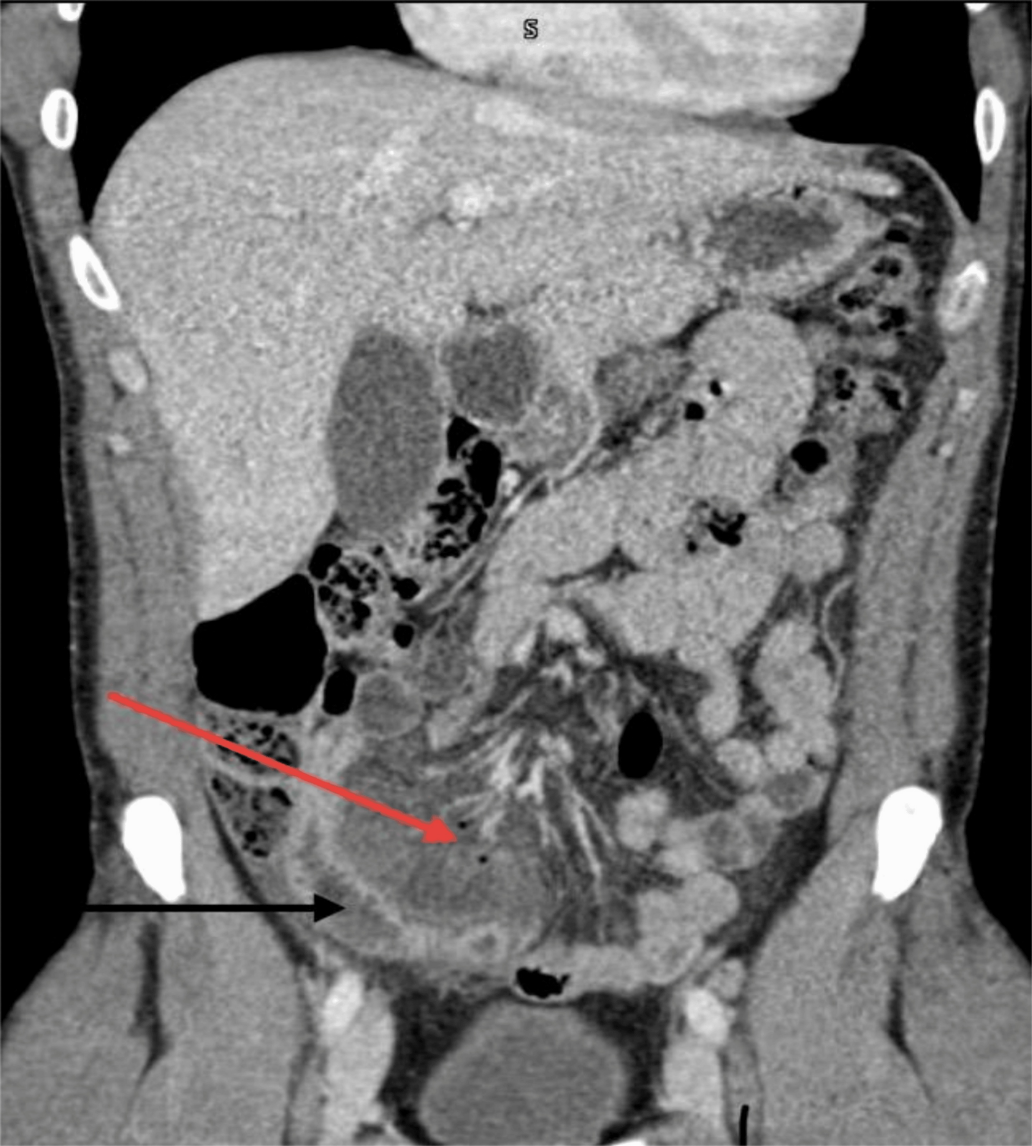
Computerized tomography of the abdomen and pelvis (coronal section) Extra-luminal air bubbles consistent with viscus perforation (red arrow); Thickened terminal ileum bowel loop wall (black arrow)

**Figure 2:**
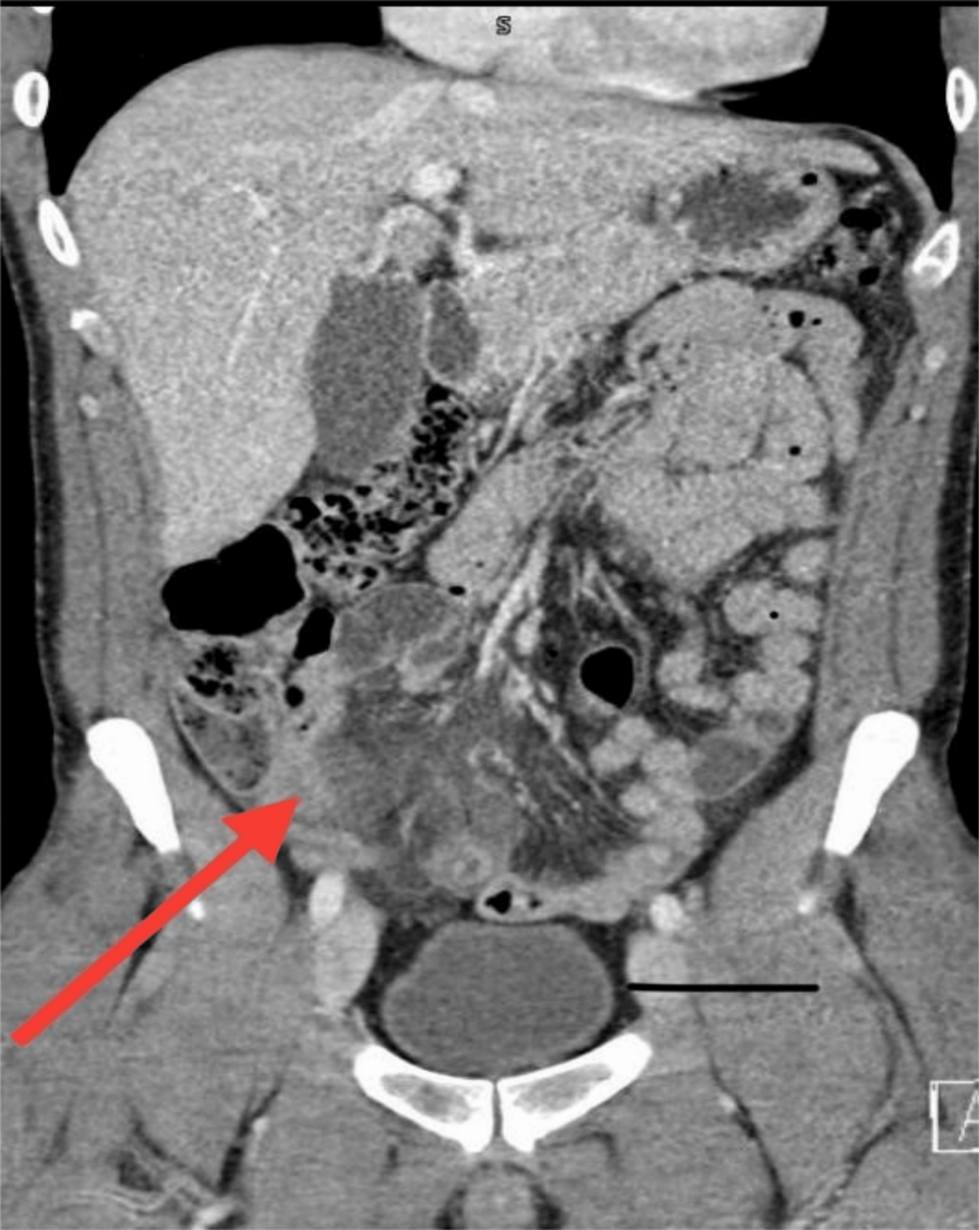
Computerized tomography of the abdomen and pelvis (coronal section) Tubular structure arising from the base of the cecum with ill-defined walls probably reflecting the dilated appendix (red arrow)

**Figure 3:**
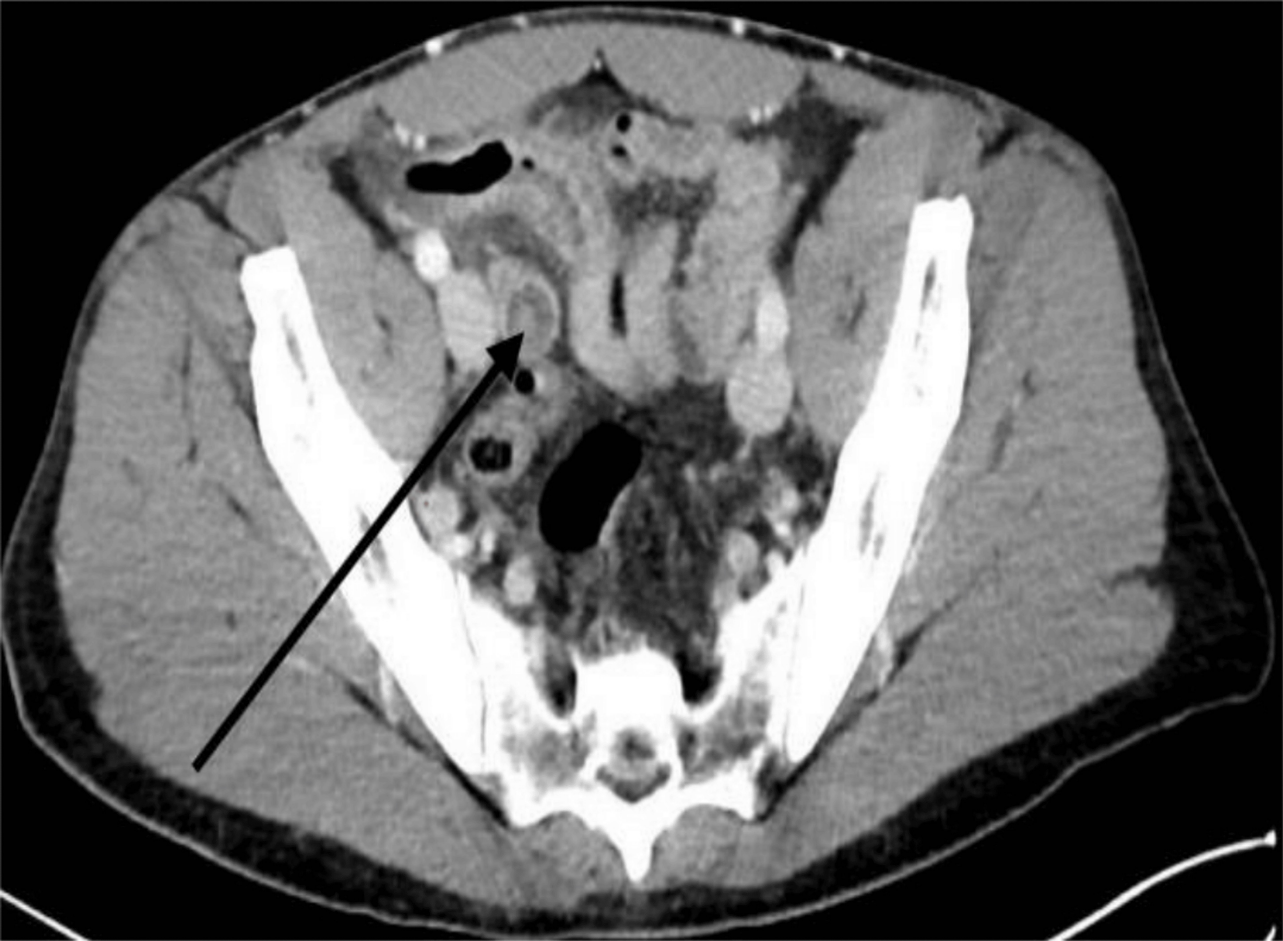
Computerized tomography of the abdomen and pelvis (axial section) Peripheral enhancing fluid collection within the right pelvis could be suspicious for abscess (black arrow)

 A 38-year-old male presented to our emergency room with an acute onset of generalized abdominal pain of a one-day duration that migrated to the right lower quadrant. His pain was associated with nausea and vomiting and he denied fever or chills. On physical exam, he was found to have right lower quadrant tenderness. Laboratory findings were within normal limits. Computerized tomography of the abdomen and pelvis was concerned for acute appendicitis with abscess formation (Figures 1-3).

**Figure 4:**
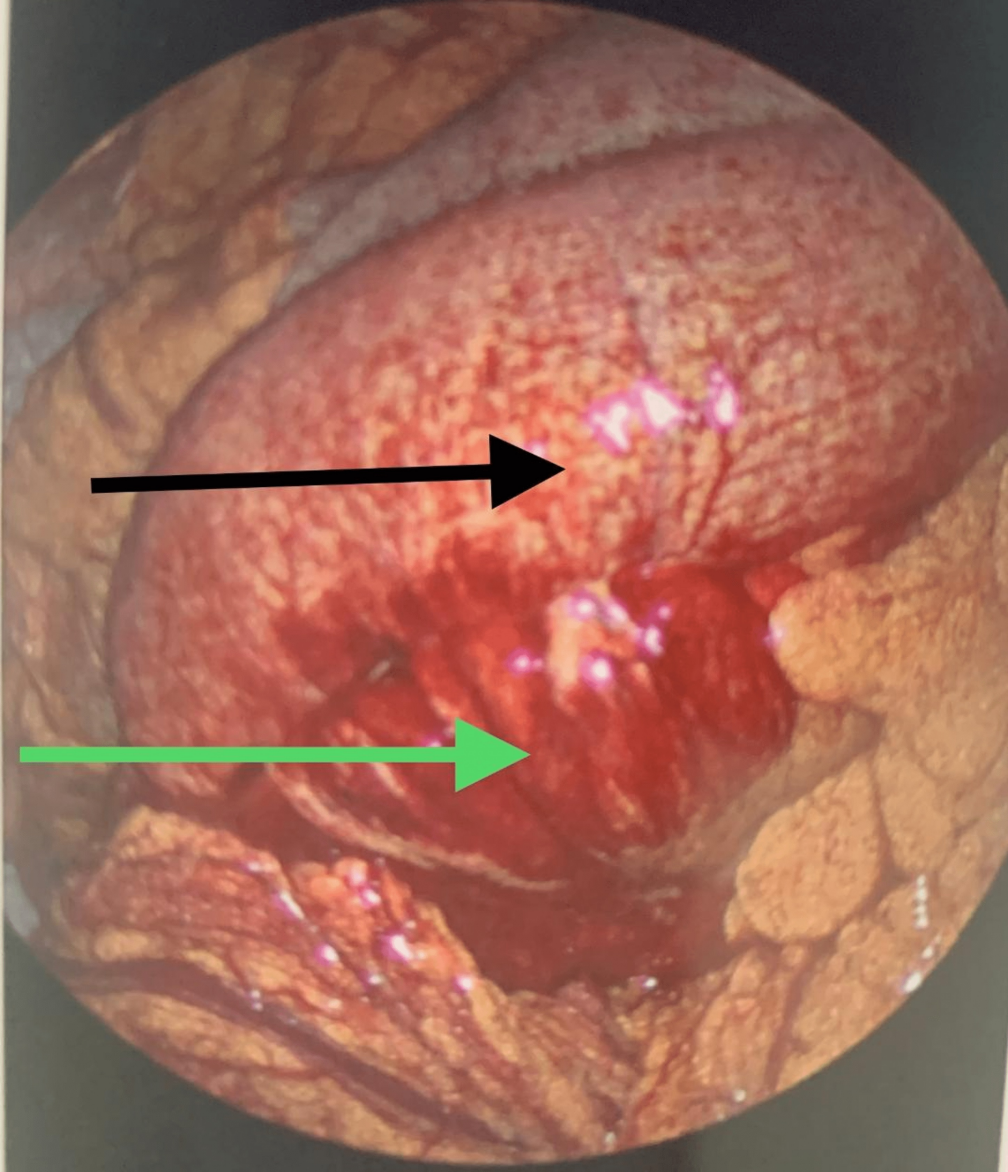
Laparoscopy findings Dilated and inflamed loop of the small bowel (black arrow); small bowel mesentery inflammation and bleeding (green arrow)

**Figure 5:**
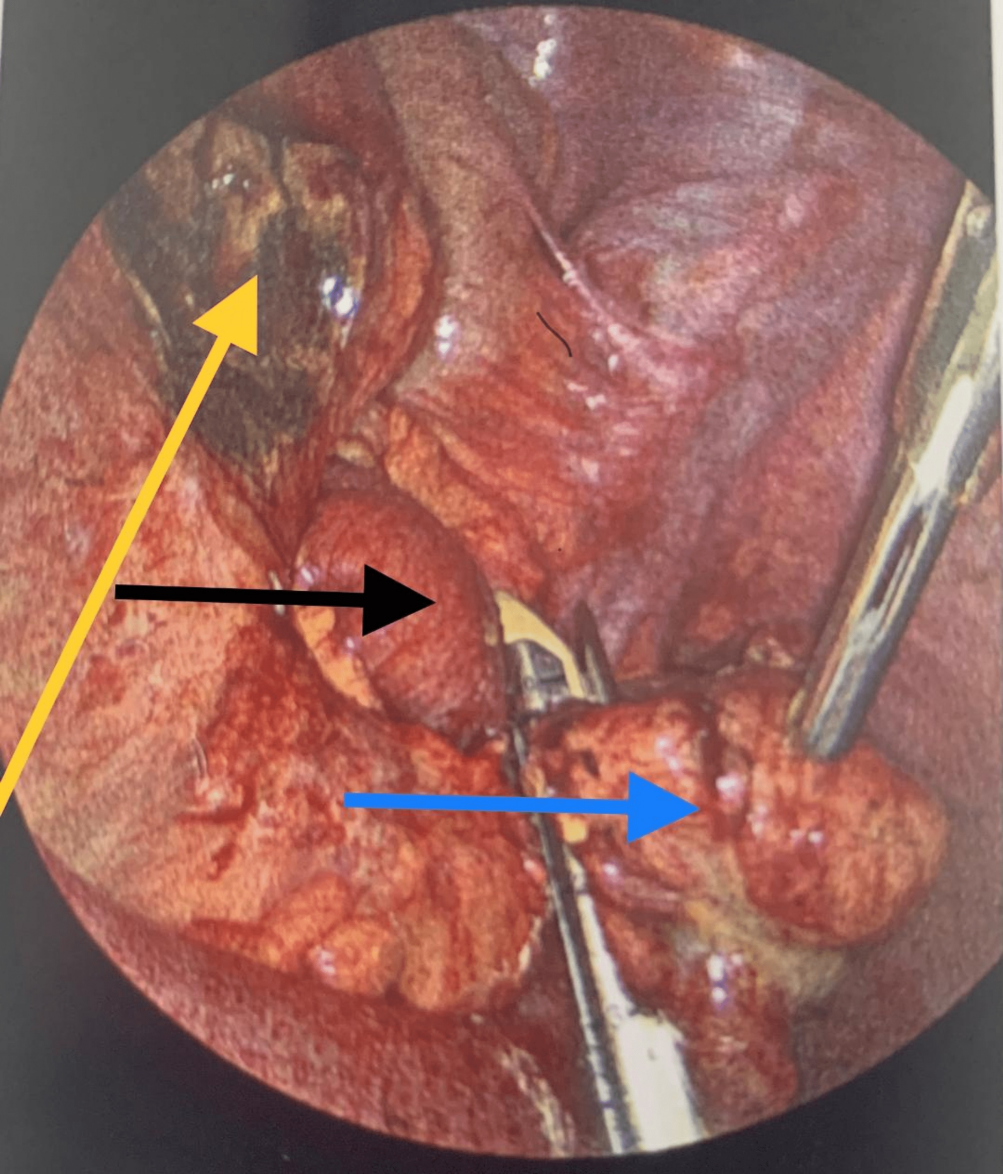
Blunt dissection of the phlegmon shown in Figure 4 revealed an abscess collection in association with perforated Meckle's diverticulum Abscess cavity after aspiration of the pus (yellow arrow); terminal ileum (black arrow); perforated Meckle's diverticulum (blue arrow)

**Figure 6:**
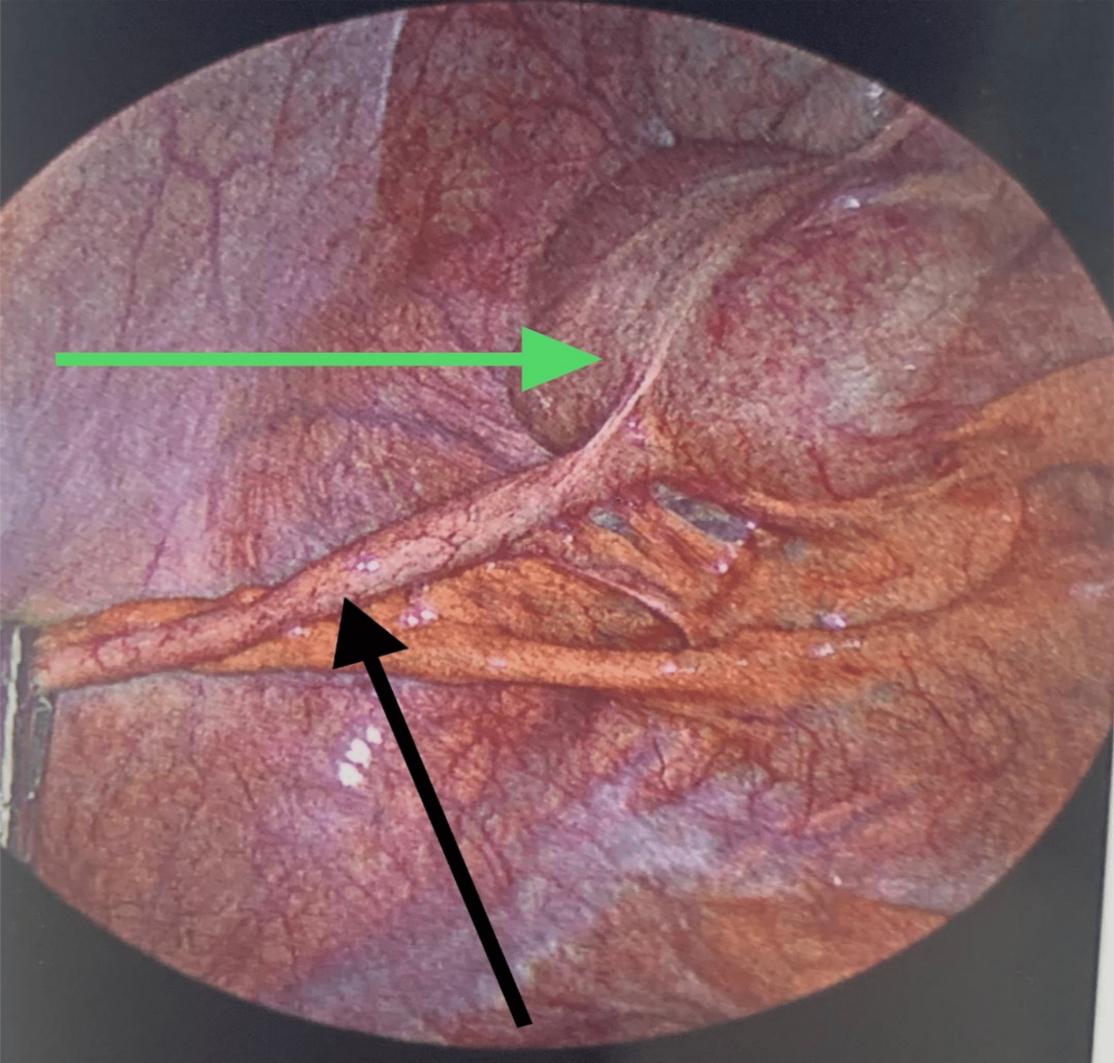
Laparoscopic view of the cecum and appendix Cecum (green arrow); Appendix (black arrow)

The patient was taken to the operating room for a laparoscopic appendectomy. Upon visualizing the normal-appearing appendix, attention was focused on the terminal ileum. A fluid collection was noted adjacent to the terminal ileum, which was aspirated and revealed frank pus. Next, attention was drawn to a perforated Meckle's diverticulum (Figures 4-6). A diverticulectomy with drain placement was performed.

The patient had an uneventful postoperative recovery and was discharged to home on postoperative day three. Pathology confirmed perforated Meckle's diverticulum with ectopic pancreatic and gastric cells.

## Discussion

MD results from incomplete obliteration of the vitelline duct that connects the primitive midgut to the yolk sac and is considered a true diverticulum, as it contains all the layers of the gastrointestinal wall [9]. It is usually incidentally discovered and present with symptoms in up to 16% of affected individuals [10]. The presence of ectopic gastric, pancreatic, duodenal, and colonic tissues dictates the symptomology and, in turn, influences the management [11]. Symptomatic MD in adults includes bowel obstruction, diverticulitis, perforation, intussusception, vesical-diverticular fistulae, and rarely malignancy [12,13]. Hemorrhage in association with peptic ulceration as a result of ectopic gastric tissue is mainly encountered in the pediatric population [14]. Symptomatic MD is difficult to diagnose, reports indicate a 5.7%-13% accurate diagnostic rate [15]. Multiple imaging modalities and techniques have been described in the diagnosis of Meckel's diverticulum. These include ultrasonography, small bowel follow-through, CT enterography, Technetium 99, double-balloon endoscopy, and magnetic resonance enterography, and capsule endoscopy [16,17]. The gold standard diagnostic modality remains Meckle's scan for children [18]. The recommended treatment for symptomatic Meckle's diverticulum is laparoscopic diverticulectomy or segmental bowel resection when the presentation is gastrointestinal bleeding with incidental diverticulectomy during surgery for other pathologies is only indicated in the pediatric population [19]. Laparoscopic resection is feasible and safe [20].

## Conclusions

Symptomatic Meckel's diverticulum can rarely present with perforation and abscess formation mimicking acute appendicitis. Computerized tomography when patients present with abdominal pain and Meckle's scan when presenting with gastrointestinal bleeding help direct the physician to the pathology location. Intraoperative attention must be placed on ruling out Meckle's diverticulum when encountered with a normal appendix. Diverticulectomy is performed when a patient presents with diverticulitis and segmental resection when gastrointestinal bleeding is the presentation (ectopic gastric cells are in the diverticulum while the bleeding ulcer is in the adjacent small bowel) and is the operative procedure of choice. An incidental finding of MD during abdominal exploration or radiological findings is not an indication for resection in adults while incidental diverticulectomy is indicated in asymptomatic children found during abdominal exploration.
